# Intelligent structure prediction and visualization analysis of non-coding RNA in osteosarcoma research

**DOI:** 10.3389/fonc.2024.1255061

**Published:** 2024-03-12

**Authors:** Longhao Chen, Liuji He, Baijie Liu, Yinghua Zhou, Lijiang Lv, Zhiguang Wang

**Affiliations:** ^1^ The Third Affiliated Hospital of Zhejiang Chinese Medical University, Hangzhou, Zhejiang, China; ^2^ The Third Clinical Medical College, Zhejiang University of Chinese Medicine, Hangzhou, Zhejiang, China; ^3^ Faculty of Orthopedics and Traumatology, Guangxi University of Chinese Medicine, Nanning, Guangxi, China; ^4^ First Affiliated Hospital, Guangxi University of Chinese Medicine, Nanning, Guangxi, China

**Keywords:** osteosarcoma (OS), non-coding RNA, visualization analysis, prediction, intelligent structure

## Abstract

**Background:**

Osteosarcoma (OS) is the most common bone malignant tumor in children and adolescents. Recent research indicates that non-coding RNAs (ncRNAs) have been associated with OS occurrence and development, with significant progress made in this field. However, there is no intelligent structure prediction and literature visualization analysis in this research field. From the perspective of intelligent knowledge structure construction and bibliometrics, this study will comprehensively review the role of countries, institutions, journals, authors, literature citation relationships and subject keywords in the field of ncRNAs in OS. Based on this analysis, we will systematically analyze the characteristics of the knowledge structure of ncRNAs in OS disease research and identify the current research hotspots and trends.

**Methods:**

The Web of Science Core Collection (WoSCC) database was searched for articles on ncRNAs in OS between 2001 and 2023. This bibliometric analysis was performed using VOSviewers, CiteSpace, and Pajek.

**Results:**

This study involved 15,631 authors from 2,631 institutions across 57 countries/regions, with a total of 3,642 papers published in 553 academic journals. China has the highest number of published papers in this research field. The main research institutions include *Nanjing Medical University* (n = 129, 3.54%), *Shanghai Jiao Tong University* (n = 128, 3.51%), *Zhengzhou University* (n = 110, 3.02%), and *China Medical University* (n = 109, 2.99%). *Oncology Letters* (n =139, 3.82%), *European Review for Medical Pharmacological Sciences* (120, 3.31%), and *Molecular Medicine Reports* (n = 95, 2.61%) are the most popular journals in this field, with *Oncotarget* being the most co-cited journal (Co-Citation = 4,268). *Wei Wang, Wei Liu*, and *Zhenfeng Duan* published the most papers, with *Wang Y* being the most co-cited author. *“miRNA”, “lncRNA”* and *“circRNA”* are the main focuses of ncRNAs in OS studies. Key themes include *“migration and invasion”, “apoptosis and proliferation”, “prognosis”, “biomarkers”* and *“chemoresistance”*. Since 2020, hotspots and trends in ncRNA research in OS include *“tumor microenvironment”, “immune”* and *“exosome”.*

**Conclusion:**

This study represents the first comprehensive bibliometric analysis of the knowledge structure and development of ncRNAs in OS. These findings highlight current research hotspots and frontier directions, offering valuable insights for future studies on the role of ncRNAs in OS

## 1 Introduction

Osteosarcoma (OS) is the most common primary malignant bone tumor, primarily affecting the rapidly growing metaphysis of long bones in children and adolescents (1). Despite advancements in surgical and pharmacological treatments, the effectiveness of late-stage OS remains unsatisfactory. The 5-year survival rate for OS patients is currently 65%, but drops to only 11-30% for those with metastatic disease, imposing significant financial burdens on patients and society (2). Understanding the mechanisms underlying the incidence, development, and metastasis of OS is crucial for identifying new diagnostic, prognostic, and therapeutic targets.

More and more evidence suggests that various non-coding RNAs (ncRNAs) play a crucial role in the occurrence and development of tumors (3). NcRNAs are a diverse group of RNA transcripts that are produced from the genome but do not become proteins (4). The majority of research on OS focuses on ncRNAs’ abnormal expression and their role in the development and spread of the tumor (3). Therefore, the expression level of ncRNAs can change in the process of disease and treatment response, and further affect the occurrence and development of OS (5).

So far, MicroRNAs (miRNAs) are the most studied short ncRNAs. Misexpression of miRNA is a common cause of occurrence, development and metastasis of many human cancers, including OS (6, 7). Some studies have proved that miRNAs may be related to bone differentiation and bone development of OS. For example, miR-23a promotes OS differentiation by down-regulating connexin-43 (Cx43/GJA1) (8), and MIR-598 regulates PDGFB and MET to play a role in osteoblast differentiation (9). Long non-coding RNAs (LncRNAs) is a kind of transcripts with more than 200 nucleotides in length, which is often involved in a variety of cellular biological processes. A large number of studies have shown that lncRNAs are abnormally regulated in OS. For example, HULC, TUG1, NEAT1 and other LncRNAs are highly expressed, while LncRNAp21, LINC-PINT, NncRNANR-136400, FER1L4 and other LncRNAs are lowly expressed (10). In addition, CircularRNAs (CircRNAs) are a new class of ncRNAs that mainly exist in mammalian cells, forming a continuous, covalently connected ring. Research has shown that CircRNAs are closely related to the occurrence and development of some human diseases, especially cancer. Qiu et al. (11). analyzed OS data in the gene expression database. They identified 15 down-regulated circRNAs, 136 up-regulated miRNAs and 52 down-regulated mRNAs, of which 14 circRNAs, 24 miRNAs and 52 mRNAs formed a circRNA-miRNA-mRNA network. It can be seen that ncRNAs has become an important part of tumor research, which provides novel and valuable insights into tumor heterogeneity and the mechanism of tumor progression and metastasis (12). However, there is a lack of complete analysis and evaluation of published literature, trends of influential countries, institutions or authors, as well as their cooperation, knowledge structure, hot topics and cutting-edge trends in ncRNA and OS disease research.

In order to determine the knowledge structure, cooperative relationships, clustering, and grouping in the literature, bibliometrics is frequently employed in qualitative and quantitative analyses of scientific publications (13, 14). The contributions of different countries, research institutions, experts and publications can be compared to present and predict the development trend of a research topic (15). Bibliometric analysis has been used in various fields of medicine, such as tumors, endocrine diseases, cardiovascular diseases, orthopedic diseases and so on (16–19). It plays an important role in analyzing hot topics, predicting frontiers and developing guidelines or knowledge graphs between ncRNAs and OS disease research.

Therefore, this study reviews the hotspots and frontier trends of ncRNAs research in OS diseases in the past 20 years by using CiteSpace and VOSviewer, and draws an intelligence structure map. This study aims to give the most recent trends, development process, frontier hotspots and trends of OS for the clinical prevention and treatment of ncRNA disorders as well as basic research.

## 2 Materials and methods

### 2.1 Search strategy

**Figure 1:**
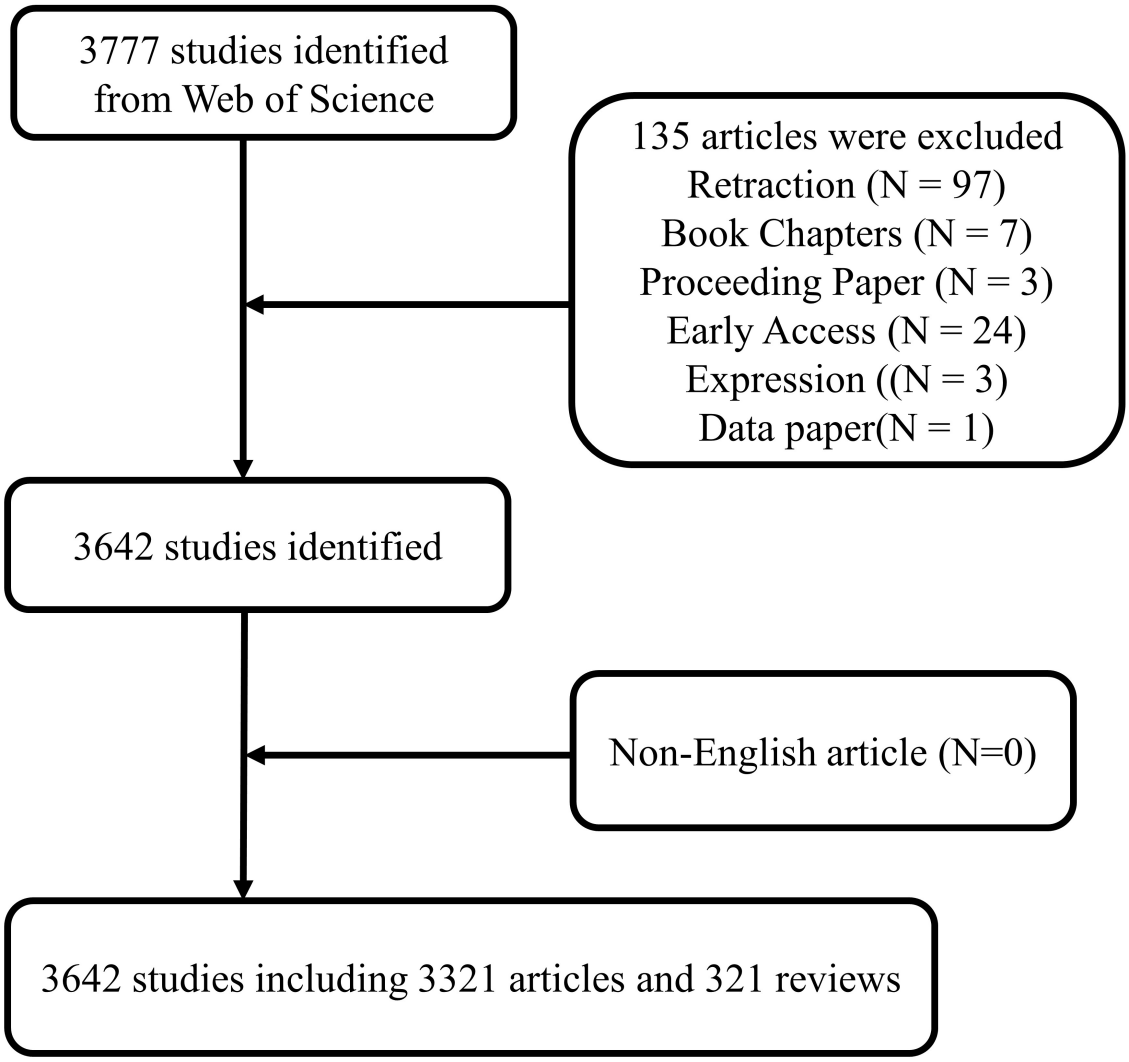
Publications screening flowchart.

We conducted a literature search on the Web of Science Core Collection (WoSCC) database (https://www.webofscience.com/wos/woscc/advanced-search) on February 8, 2024. The search formula is ((TS = (Osteosarcom)) AND TS = ((“lncRNA” OR “lnc RNA” OR “long ncRNA” OR “long noncoding RNA” OR “long non coding RNA” OR “long non translated RNA” OR “long non protein coding RNA” OR “linc RNA” OR “lincRNA” OR “microRNA” OR “micro RNA” OR “mi RNA” OR “miR” OR “circRNA” OR “circ RNA” OR “circular RNA” OR “circular noncoding RNA” OR “circular non coding RNA” OR “circular ncRNA” OR “circular ncRNA” OR “circular nonprotein coding RNA” OR “circular nontranslated RNA” OR “circular untranslated RNA” OR “ribosomal RNA” OR “rRNA” OR “transfer RNA” OR “tRNA” OR “tRNA-derived small RNAs” OR “tsRNA” OR “Piwi-interacting RNA” OR “PiRNA” OR “small nucleolar RNA” OR “snoRNA” OR “small nuclear RNA” OR “snRNA” OR “tRNA-Derived Fragments” OR “tRF” OR “tRNA halves” OR “tiRNA” OR “small interfering RNA” OR “siRNA” OR “small cytoplasmic RNA” OR “scRNA”)) AND LA = (English) AND DOP = (2001-01-01/2023-12-31)AND DT = (Article OR Review) (**Figure 1**).

### 2.2 Data analysis

Software for bibliometrics analysis, VOSViewer (version 1.6.20), is used to extract data and information from the literature (20). The size, color, and line thickness of each node in the VOS viewer-generated chart correspond to the quantity and classification of the items they represent, respectively, and the degree of collaboration or co-citation between them. Each node represents a project (21, 22). We use it to establish a network of cooperation, co-citation and co-occurrence (23, 24). In our research, the software mainly completes the following analysis: national and institutional analysis, journal and co-citation journal analysis, author and co-citation author analysis, keyword co-occurrence analysis.

Another program created by Professors Chen C for bibliometric analysis and visualization is CiteSpace (version 6.2.R6) (25). CiteSpace was used in our study to map the journals’ dual-map overlay and to evaluate references using Citation Bursts.

According to the Journal Citation report 2022, we provide a summary of the periodical division and Influence factors. In addition, a quantitative analysis of the publication is performed using Microsoft Office Excel 2021.

## 3 Results

### 3.1 Quantitative analysis of publication

**Figure 2:**
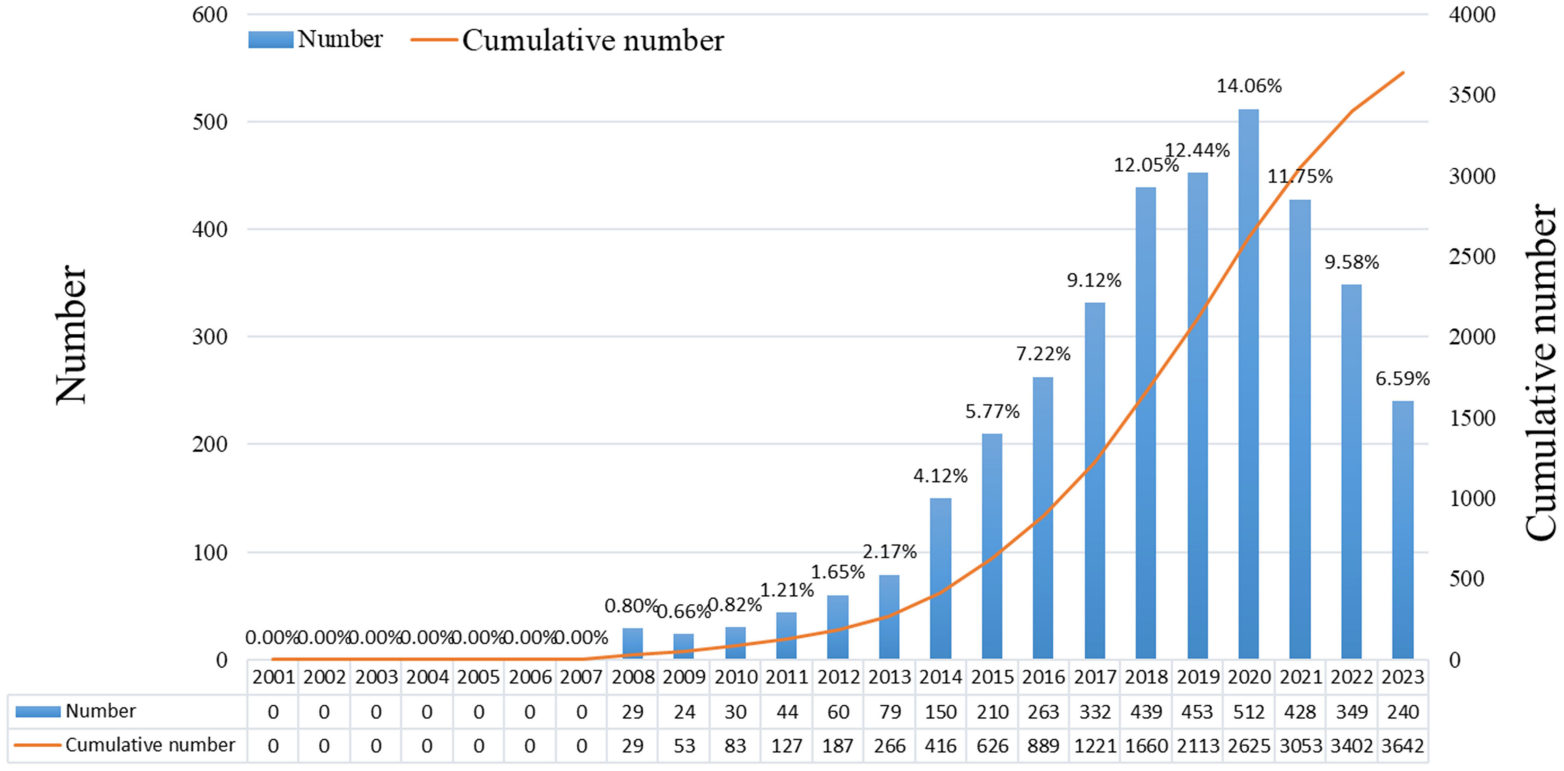
Number of annual publications on the role of ncRNA in OS research in 2001-2021.

There were 3,642 studies on ncRNAs of OS in the past 23 years, including 3,321 “articles” and 321 “reviews”. As shown in **Figure 2**, the number of publications was 0 before 2008, and there were no related research publications. Since 2014 the number of posts has increased rapidly. The number of publications in 2020 was 512, 2.0 times that of 2016. From the trend chart, we can see that the research on OS ncRNAs are mainly concentrated in the past 10 years, reaching a peak at the end of 2020. The number of publications declined in 2021-2023. The specific reason for the decline is not very clear, preliminary speculation may be related to the outbreak of COVID-19. In addition, the cumulative number of articles in the past 23 years showed an upward trend.

### 3.2 Country and institutional analysis

**Figure 3:**
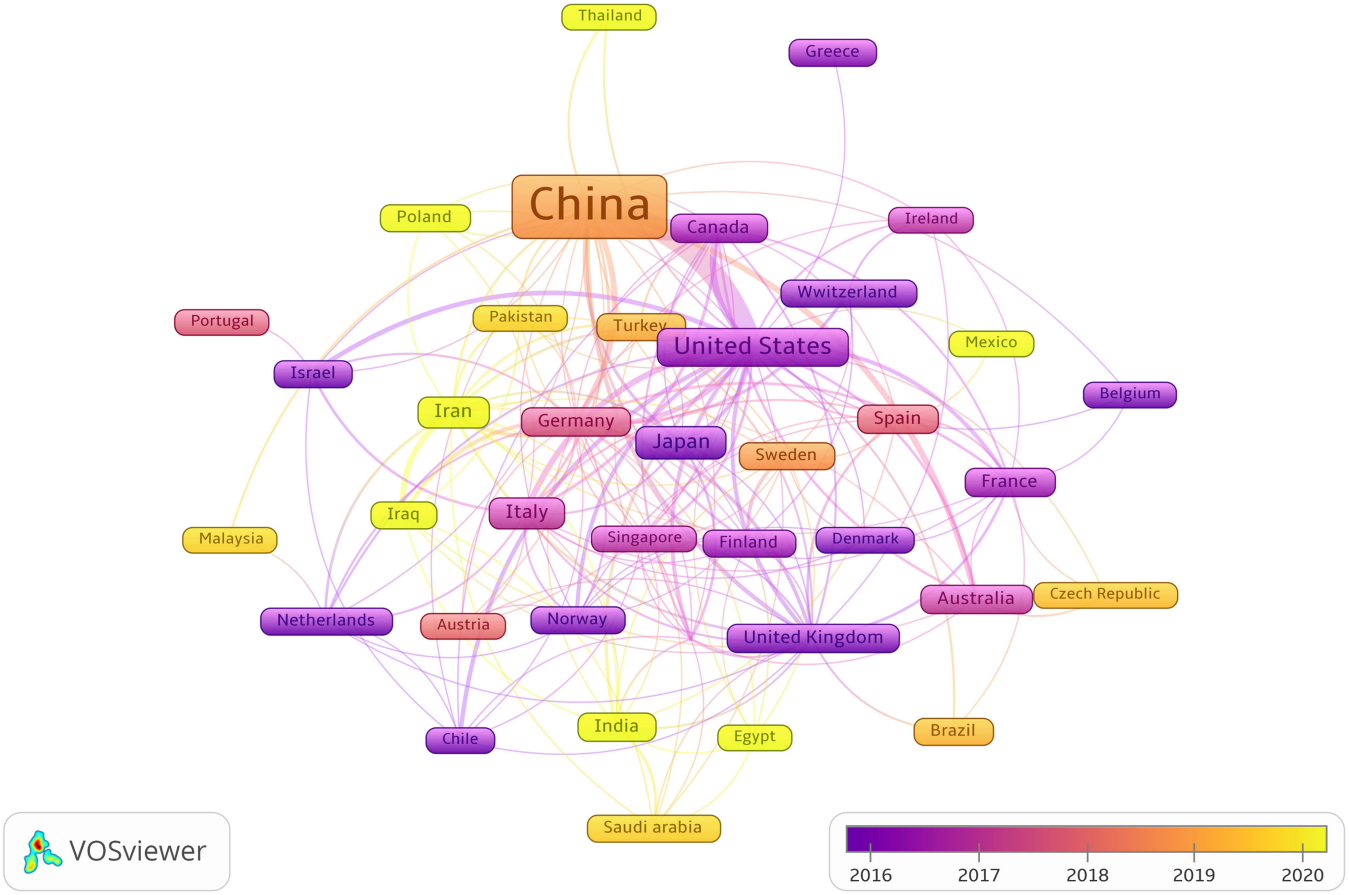
The visualization of cooperation networks between countries involved on the roles of ncRNAs in OS research. (Each node represents a different country, and the node size represents the number of papers published. The connection between the nodes indicates the cooperation relationship, and the thickness of the lines indicates the cooperation intensity. The color of the node represents the average year).

Publications from 57 countries and 2,631 institutions were included in the study. The distribution of the top ten countries is shown in **Table 1**. Among these countries, China has the largest number of publications (n = 3,084, 84.68%), followed by the United States (n = 289, 7.94%). Then, depending on the quantity of publications in each nation, we created a collaborative network using a visual analysis of 38 countries related to publications (**Figure 3**). It is important to note that there will be variations in the level of collaboration between various nations. For example, China has close cooperation with United States, Germany, Canada, United Kingdom and Australia. The United States also actively cooperates with Israel, Chile, Canada and Italy. From the perspective of time scale, the average number of papers published in the United States, United Kingdom, Germany and Singapore are mainly concentrated in 2016. The research in China, Turkey, Sweden and Austria are mainly concentrated in 2018, which started relatively late.

**Table 1:** Top 10 countries and institutions on the roles of ncRNAs in OS research.

Rank	Country/Regions	Counts (%)	Institutions	Counts (%)
1	China	3,084 (84.68%)	Nanjing Medical University (China)	129 (3.54%)
2	United States	289 (7.94%)	Shanghai Jiao Tong University (China)	128 (3.51%)
3	Japan	88 (2.42%)	Zhengzhou University (China)	110 (3.02%)
4	Italy	62 (1.70%)	China Medical University (China)	109 (2.99%)
5	Iran	54 (1.48%)	Jilin University (China)	109 (2.99%)
6	Germany	45 (1.24%)	Shandong University (China)	94 (2.58%)
7	Australia	38 (1.04%)	Harbin Medical University (China)	88 (2.42%)
8	United Kingdom	36 (0.99%)	Wuhan University (China)	86 (2.36%)
9	South Korea	34 (0.93%)	Central South University (China)	83 (2.28%)
10	Canada	33 (0.91%)	Southern Medical University (China)	82 (2.25%)

**Figure 4:**
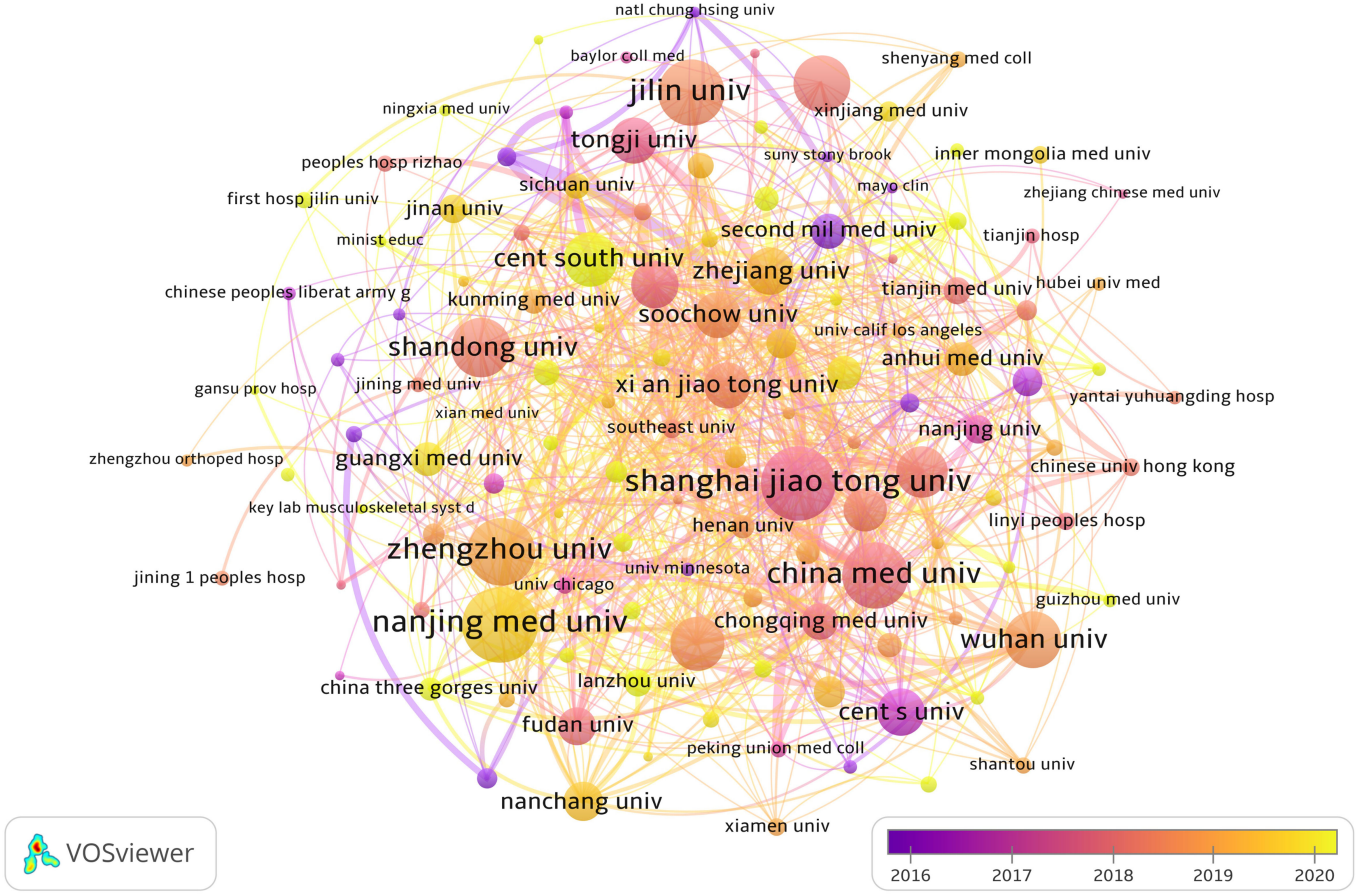
The visualization of institutions involved in the roles of ncRNA in OS research. (Each node represents a different Institutional, and the node size represents the number of papers published. The connection between the nodes indicates the cooperation relationship, and the thickness of the lines indicates the cooperation intensity. The color of the node represents the average year).

From **Table 1**, we can see that Chinese research institutions publish the most research results. The four institutions with the largest number of publications are: *Nanjing Medical University* (129, 3.54%), *Shanghai Jiao Tong University* (128, 3.51%), *Zhengzhou University* (110, 3.02%) and *China Medical University* (109, 2.99%). Then, we set the screening criteria to analyze 125 institutions and build a cooperative network according to the number of publications and partnerships of each institution (**Figure 4**). As shown in **Figure 4**, there is a close cooperative relationship among *Shanghai Jiao Tong University, Tongji University* and *Fudan University.* The *Massachusetts General Hospital*, *Harvard University*, *Huazhong University of Science and Technology, and Zhengzhou University* also cooperate actively.

### 3.3 Journals and co-cited journals

**Figure 5:**
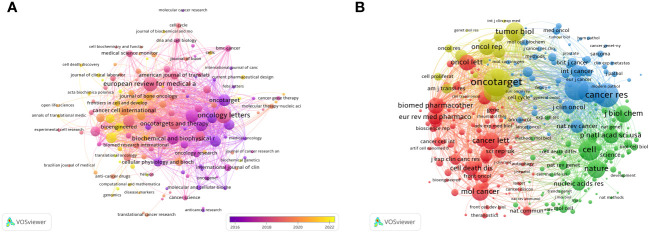
The visualization of journals **(A)** and co-cited journals **(B)** involved in the roles of ncRNA in OS development research. [**(A)** Each node represents a different journal, and the node size represents the number of papers published. The connection between the nodes indicates the cooperation relationship, and the thickness of the lines indicates the cooperation intensity. The color of the node represents the average year. [**(B)** Each node represents a different co-cited journals, and the node size represents the total number of citations. The connection between the nodes indicates the relevance between the co-cited journals, and the line thickness indicates the stronger the relevance. The color of the node represents the clustering relationship].

After screening the literature, we found that the papers on ncRNAs in OS were published in 553 journals. *Oncology Letters* published most of the journals (n = 139, 3.82%), followed by *European Review for Medical and Pharmacological Sciences* (120, 3.31%) and *Molecular Medicine Reports* (n = 95, 2.61%) (**Table 2**). Among these journals, the journal with the highest impact factor is *Biomedicine & Pharmacotherapy* (IF = 7.5), followed by *Cancer Cell International* (IF = 5.8) (**Table 2**). Then, we selected 140 journals to draw a journal network (set the minimum number of publications to be 5). As shown in **Figure 5A**, *Oncology letters* has an active citation relationship with journals such as *European Review, Medical on oncology, Cellular physiology and biochemistry* and so on.

**Table 2:** Top 15 Journals and co-cited journals on research of ncRNAs in OS research.

Rank	Journal	Count(%)	IF (2022)	JCR	Co-Cited Journal	Co-Citation	IF (2022)	JCR
1	Oncology Letters	139 (3.82%)	2.9	Q3	Oncotarget	4,268	/	/
2	European Review for Medical and Pharmacological Sciences	120 (3.31%)	3.3	Q2	Cancer Research	3,500	11.2	Q1
3	Molecular Medicine Reports	95 (2.61%)	3.4	Q3	Plos One	3,378	3.7	Q2
4	Oncotargets and Therapy	82 (2.25%)	4.0	Q2	Cell	2,793	64.5	Q1
5	Biochemical and Biophysical Research Communications	81 (2.22%)	3.1	Q2	Oncogene	2,737	8.0	Q1
6	Oncotarget	81 (2.22%)	/	/	Biochemical and Biophysical Research Communications	2,712	3.1	Q2
7	Biomedicine & Pharmacotherapy	74 (2.03%)	7.5	Q1	Tumor Biology	2,410	/	/
8	Oncology Reports	73 (2.00%)	4.2	Q2	Nature	2,244	64.8	Q1
9	Tumor Biology	69 (1.89%)	/	/	Cancer Letters	2,043	9.7	Q1
10	Frontiers in Oncology	65 (1.78%)	4.7	Q2	Molecular Cancer	2,040	37.3	Q1
11	International Journal of Clinical and Experimental Pathology	63 (1.73%)	/	/	Journal of Biological Chemistry	2,035	4.8	Q2
12	Journal of Cellular Biochemistry	57 (1.57%)	4.0	Q2	Oncology Letters	1,823	2.9	Q4
13	Cancer Cell International	55 (1.51%)	5.8	Q1	Oncology Reports	1,801	4.2	Q2
14	Plos One	54 (1.48%)	3.7	Q2	Biomedicine & Pharmacotherapy	1,788	7.5	Q1
15	Experimental and Therapeutic Medicine	52 (1.43%)	2.7	Q3	Proceedings of the National Academy of Sciences of The United States Of America	1,774	11.1	Q1

There are 3 journals that have been cited more than 3,000 times (**Table 2**). *Oncotarget* (Co-Citation = 4,268) is the most frequently cited journal, followed by *Cancer Research* (Co-Citation = 3,500) and Plos One (Co-Citation = 3,378). In addition, the influencing factor of *Nature* was the highest (IF = 64.8), followed by *Cell* (IF = 64.5). As shown in **Figure 5**, we draw a co-citation network diagram after screening (set the number of citations to at least 80) (**Figure 5B**). *Oncotarget* has a positive co-citation relationship with *Tumor Biology, Cancer Research, Oncology Letters and Molecular Cancer. Cell* has a positive co-citation relationship with *Nature*, *Cancer Research, Molecular Cancer Plos one and so on.*

**Figure 6:**
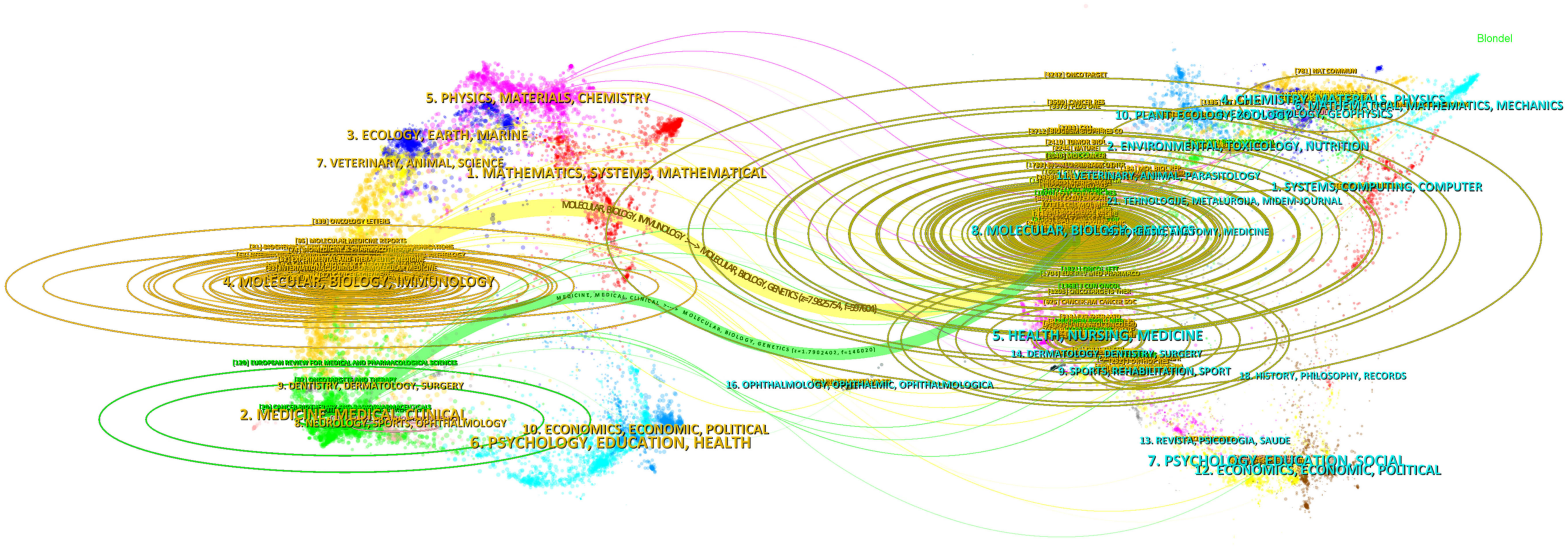
The dual-map overlay of journals involved in the roles of ncRNA in OS research. (On the left is the citation periodical group, and on the right is the cited periodical group. The yellow and green lines are thicker, indicating that they are the main citation paths).

The double map superposition of periodicals can see the common research hotspots in the two fields, so we use the double map superposition of periodicals to show the citation relationship between journals and co-cited journals. On the left is the citation journal clustering, and the cited journal clustering is on the right (26). In **Figure 6**, the yellow line is thicker, indicating that it is the main citation path, which represents the research of ncRNAs in OS is published in Molecular/Biology/immunology journals mainly cited by Molecular/Biology/Genetics journals, of which *Oncotarget* is the most frequently cited. In addition, another green path indicates that the research of OS ncRNAs published in Molecular/Medicine/Medical/Clinical journals cited by Molecular/Biology/Genetics journals.

### 3.4 Authors and Co-cited authors

**Figure 7:**
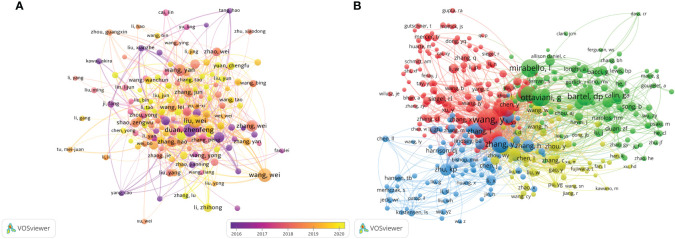
The visualization of authors **(A)** and co-cited authors **(B)** involved in the roles of ncRNAs in OS development research. [**(A)** Each node represents a different author, and the node size represents the number of papers published. The connection between the nodes indicates the cooperation relationship, and the thickness of the lines indicates the cooperation intensity. The color of the node represents the average year. [**(B)** Each node represents a different co-cited author, and the node size represents the total number of citations. The connection between the nodes indicates the relevance between the co-cited author, and the line thickness indicates the stronger the relevance. The color of the node represents the clustering relationship].

We extracted the author information from the literature and found that a total of 15,631 authors participated in the study of ncRNA in OS. The top three authors have published more than 30 articles (**Table 3**). We have established a cooperative network (set the number of published papers to be greater than or equal to 7 (**Figure 7A**). We have observed some partnerships. For example, *Wei Liu, Wei Zhang, Bin Wang, Kun Wang, Yang Cao, Yang Wang, etc* have close cooperation. *Wei Zhang, Hao Li, Ming Liu, Yi Liu, Yong Zhang, Lu Zhang* actively cooperates.

**Table 3:** Top 10 authors and co-cited authors on research of ncRNAs in OS.

Rank	Authors	Count	Co-cited Authors	Citations
1	Wang, Wei	33	Wang, Y	676
2	Liu, Wei	32	Bartel, DP	488
3	Duan, Zhenfeng	31	Mirabello, I	474
4	Wang, Yan	27	Ottaviani, G	453
5	Zhang, Wei	25	Zhang, Y	431
6	Hornicek, Francis j.	22	Livak, KJ	373
7	Zhang, Hao	21	Zhang, J	357
8	Zhang, Jun	20	Li, J	353
9	Wang, Lei	19	Li, Z	327
10	Zhang, Lei	19	Chen, X	319

There are 49,777 co-cited authors. Among the top 10 co-cited authors, 5 authors have been cited more than 400 times (**Table 3**). The most co-cited author is *Wang Y* (n = 676), followed by *Bartel DP* (n = 488), *Mirabello I* (n = 474), *Ottaviani G* (n = 453) and *Zhang Y* (n = 431). We set the minimum number of co-citations equal to 40 to screen co-cited authors, and then draw a co-citation network diagram. (**Figure 7B**). As shown in **Figure 7B**, *Wang Y, Zhu KB, Li ZW* and other authors have a positive cooperative relationship.

### 3.5 Co-cited references

**Figure 8:**
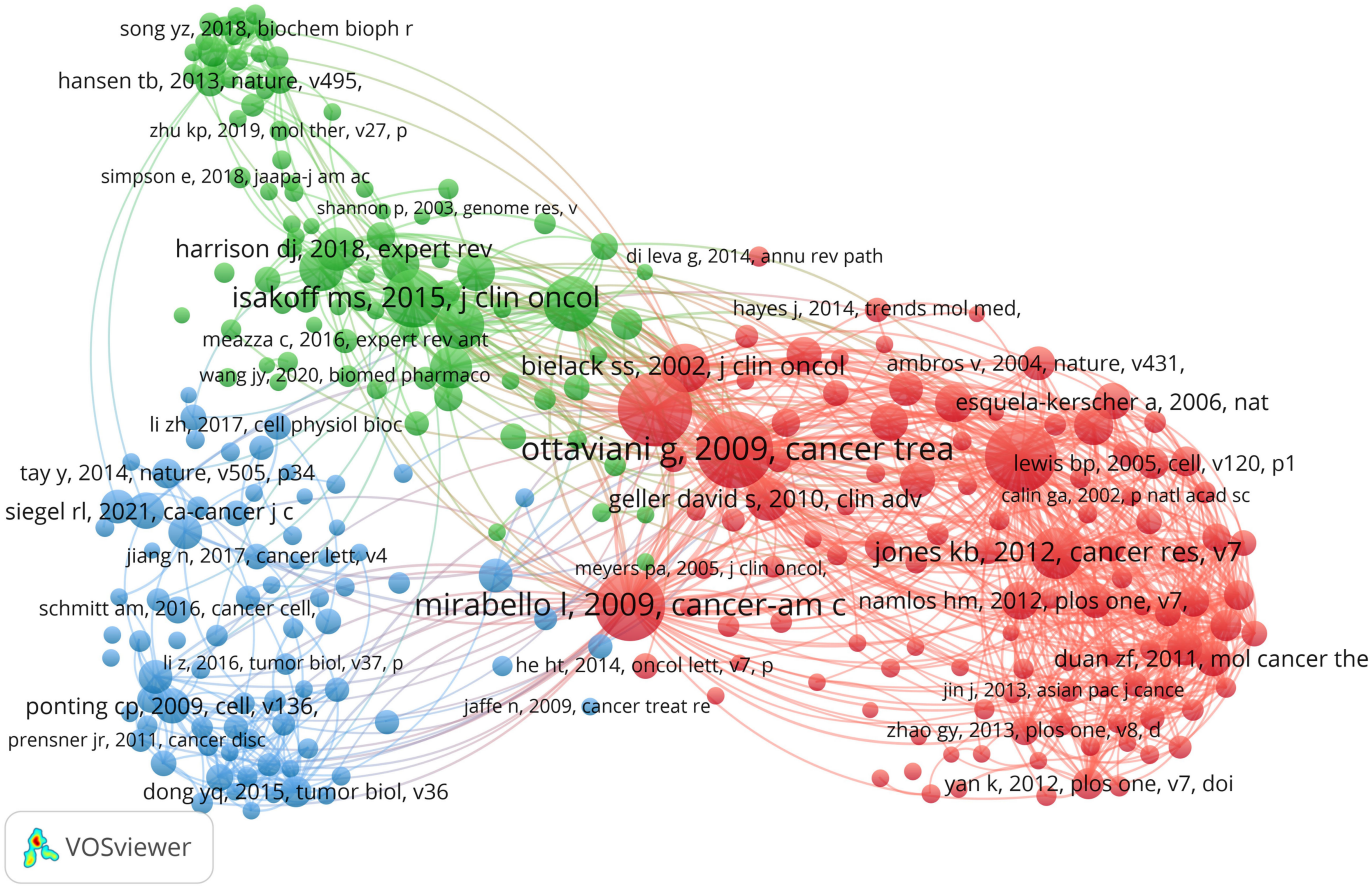
The visualization of co-cited references on the roles of ncRNAs in OS research. (Each node represents a different co-cited reference, and the node size represents the total number of citations. The connection between the nodes indicates the relevance between the cited reference, and the line thickness indicates the stronger the relevance. The color of the node represents the cluster relationship).

Over the past decade, there have been 85,361 co-cited references on ncRNA studies in OS. The top 10 co-cited references listed in **Table 4** have all received at least 160 co-citations. We have constructed a co-citation network diagram (setting the number of co-citations greater than or equal to 30) (**Figure 8**). “*Ottaviani G, 2009, Cancer Treatment and Research*” shows active co-cited relationships with “*Mirabello I, 2009, Cancer*”, “*Bartel DP, 2004, Cell*”, *“Jones KB, 2012, Cancer Research”*, etc.

**Table 4:** Top 10 co-cited references on the roles of ncRNAs in OS research.

Rank	First Author	Journal	IF (2022)	JCR	DOI	Citations
1	Ottaviani G	Cancer Treatment and Research	/	/	10.1007/978-1-4419-0284-9_1	395
2	Livak KJ	Methods	4.8	Q1	10.1006/meth.2001.1262	373
3	Bartel DP	cell	64.5	Q1	10.1016/s0092-8674 (04) 00045-5	353
4	Mirabello I	Cancer	6.2	Q1	10.1002/cncr.24121	338
5	Isakoff MS	Journal of Clinical Oncology	45.3	Q1	10.1200/jco.2014.59.4895	245
6	Luetke A	Cancer Treatment Reviews	11.8	Q1	10.1016/j.ctrv.2013.11.006	230
7	Jones KB	Cancer Research	11.2	Q1	10.1158/0008-5472.can-11-2663	196
8	Bielack SS	Journal of Clinical Oncology	45.3	Q1	10.1200/jco.20.3.776	186
9	Kansara M	Nature Reviews Cancer	78.5	Q1	10.1038/nrc3838	182
10	Ritter J	Annals of Oncology	50.5	Q1	10.1093/annonc/mdq276	160

### 3.6 Reference with citation bursts

**Figure 9:**
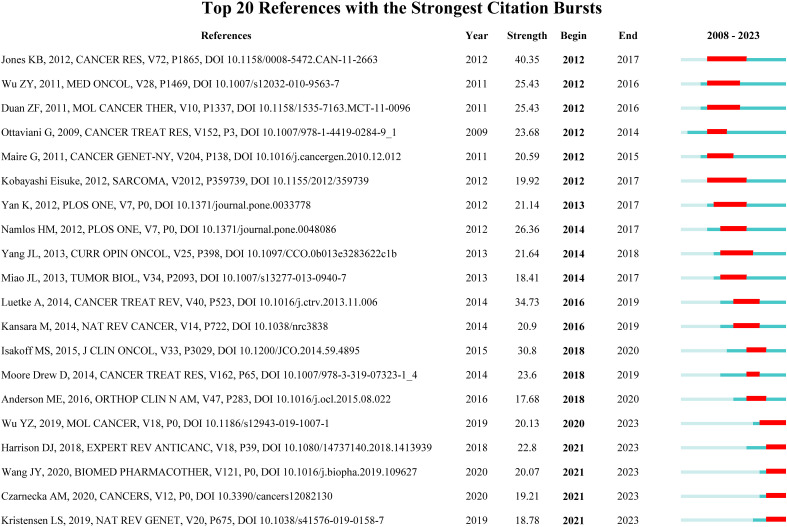
Top 20 References with the Strongest Citation Bursts. (The blue line represents the overall time range of 2008-2023. Red lines indicate a strong abrupt citation over a year or several years).

Reference with citation bursts refers to the literature that is often cited in this field for a period of time. In our study, CiteSpace identified 20 references with strongly cited outbreaks (**Figure 9**). Our research shows that citation bursts for references occurred as early as 2008 and lasted until 2023. As shown in **Figure 9**, Red lines indicate a strong abrupt citation over a year or several years (27). The burst intensity range of the 20 references was 17.68 to 40.35, and the endurance intensity was between 2 and 5 years. The contents of **Table 5** are the main research contents of the top 20 citations Bursts of reference. The outbreak of the strongest citations (strength = 40.35) was titled “*Mechanism of chemoresistance mediated by miR-140 in human osteosarcoma and colon cancer cells*”, authored by *Jones KB et al.* with citation bursts from 2012 to 2017. The second ranked reference in the citation burst (strength = 34.73) was titled “*Osteosarcoma treatment - where do we stand? A state of the art review*”, published in *Cancer Treatment Reviews* by *Luetke A et al.* with citation bursts from 2016 to 2019.

**Table 5:** The main research contents of the 20 references with strong citations bursts.

Rank	Strength	Main research content
1	40.35	Mechanism of chemoresistance mediated by miR-140 in human osteosarcoma and colon cancer cells
2	34.73	Osteosarcoma treatment - where do we stand? A state of the art review
3	30.8	Osteosarcoma: Current Treatment and a Collaborative Pathway to Success
4	26.36	Modulation of the osteosarcoma expression phenotype by microRNA
5	25.43	MicroRNA-21 is involved in osteosarcoma cell invasion and migration
6	25.43	MicroRNA-199a-3p is downregulated in human osteosarcoma and regulates cell proliferation and migration
7	23.68	The epidemiology of osteosarcoma
8	23.6	Osteosarcoma:This chapter will highlight the clinical presentation, diagnosis, and treatment of osteosarcoma.
9	22.8	Current and future therapeutic approaches for osteosarcoma
10	21.64	New molecular insights into osteosarcoma targeted therapy
11	21.14	MicroRNA-34a inhibits the proliferation and metastasis of osteosarcoma cells both *in vitro* and *in vivo*
12	20.9	Translational biology of osteosarcom
13	20.59	Analysis of miRNA-gene expression-genomic profiles reveals complex mechanisms of microRNA deregulation in osteosarcoma
14	20.13	Circular RNA circTADA2A promotes osteosarcoma progression and metastasis by sponging miR-203a-3p and regulating CREB3 expression
15	20.07	Potential regulatory role of lncRNA-miRNA-mRNA axis in osteosarcoma
16	19.92	MicroRNA Involvement in Osteosarcoma
18	18.78	The biogenesis, biology and characterization of circular RNAs
19	18.41	MicroRNAs in osteosarcoma: diagnostic and therapeutic aspects
20	17.68	Update on Survival in Osteosarcoma

### 3.7 Hotspots and frontiers

Analyzing keyword co-occurrences is crucial for identifying research hotspots in a certain area. **Table 6** shows the top 40 high-frequency keywords. Among these keywords, except for the subject words we searched, *migration and invasion, apoptosis/proliferation, biomarker, chemoresistance, epithelial-mesenchymal transition and wnt/beta-catenin pathway* appear more than 100 times, which represent the main hot research direction of ncRNAs in OS. Among the keywords related to ncRNAs, miRNA has the most keywords (n = 1,965), followed by lncRNA (n = 644) and circRNA (n = 255).

**Table 6:** Top 20 keywords on research of ncRNAs in OS.

Rank	Keywords	Counts	Rank	Keywords	Counts
1	osteosarcoma	2,267	21	exosome	61
2	mirna	1,965	22	cell cycle	56
3	apoptosis/proliferation	752	23	diagnosis	56
4	migration and invasion	750	24	immune	55
5	lncrna	644	25	tumor suppressor	54
6	prognosis	269	26	sirna	53
7	circrna	255	27	gene expression	50
8	biomarker	174	28	cisplatin	48
9	chemoresistance	169	29	mmp-	48
10	epithelial-mesenchymal transition	116	30	sox-	43
11	wnt/beta-catenin pathway	103	31	p53	42
12	pi3k/akt signal pathway	86	32	snhg-	39
13	lung metastasis/lung cancer	85	33	angiogenesis	36
14	autophagy	75	34	bioinformatics	36
15	target therapy	73	35	meta-analysis	35
16	mesenchymal stem cell	66	36	vegf	35
17	cerna	65	37	tumor microenvironment	34
18	chemosensitivity	65	38	nf-kb signal pathway	33
19	survival	65	39	pten	33
20	progression	63	40	oncogene	32

**Figure 10:**
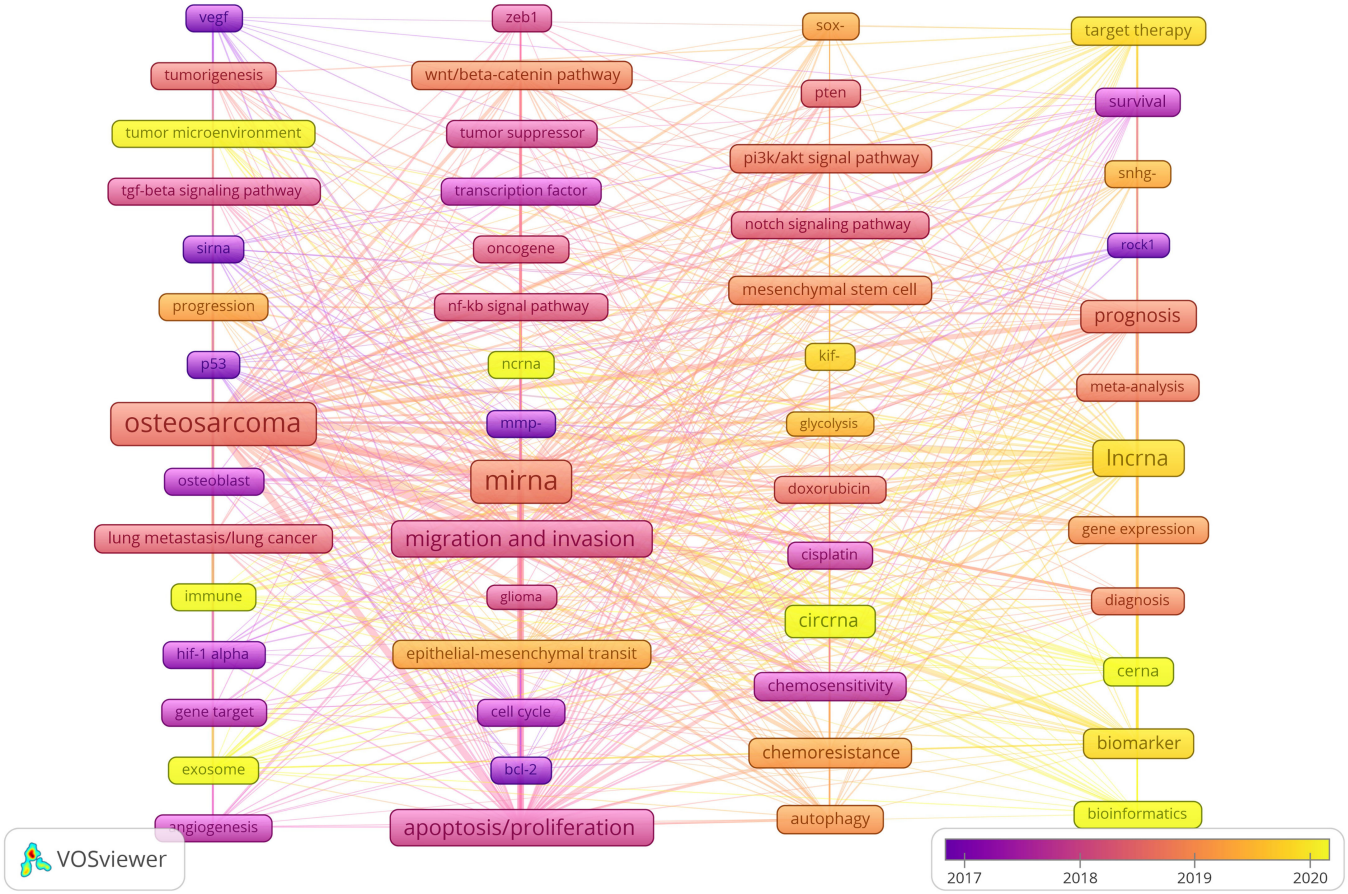
Keyword cluster analysis and the distribution characteristics of average publication years. (Each node represents a different Keyword, and the node size represents the frequency of keyword occurrence. The connection between the nodes indicates the association, and the line thickness indicates the association strength. The color of each node shows the average appearing year of the keyword).

We use lines to connect nodes, the thicker the line, the closer the relationship, and the larger the node, the more frequency. As shown in **Figure 10**, we analyze the information of keywords and obtain four clusters. For example, the keywords in the first clusters consists of angiogenesis “*vegf”, “tumorigenesis”, “tumor microenvironment”, “tgf-beta signaling pathway”, “sirna, progression”, “p53”, “osteosarcoma”, “osteoblast”, “lung metastasis/lung cancer”, “immune”, “hif-1 alpha”, “gene target”, “exosome”*, etc. From the distribution characteristics of keywords in the average publication year, the research on ncRNAs of OS is mainly concentrated in 2017-2020. Among them, the research on siRNAs were earlier, concentrated around 2017, the research on miRNAs were concentrated around 2018-2019, the research on lncRNAs were concentrated around 2019-2020, the research on circRNAs were later, concentrated in 2020. In addition, we also found that the research of “*tumor microenvironment”, “immune”, “exosome”, “cerna” and “bioinformatics”* in ncRNA is mainly concentrated in 2020 or later, indicating that they are the research hotspots and trends since 2020.

## 4 Discussion

### 4.1 General information

We compile the available literature by year and discover that there was zero material published between 2001 and 2007, proving that the current research on ncRNA in OS has not been conducted and that there is no scientific foundation for this topic. An average of 44 publications were published annually between 2008 and 2013, showing that this area of study is still in its early stages. From 2014 to 2023, the number of papers published began to increase significantly. In the past five years, the research publications on ncRNA in OS have exploded, indicating that more and more scholars have paid attention to this research.

Judging from the publications and cooperative relations of different countries, China is the main force in carrying out ncRNAs research on OS, followed by the United States. We have noticed that there are varying degrees of cooperation among China, United States, Germany, Canada, United Kingdom and Australia. In addition, the United States is actively cooperating with Israel, Chile, Canada and Italy. From the point of view of the research time and the average number of published literature, the research on ncRNAs in OS in the United States, Japan, Switzerland, United kingdom, Canada, Israel, Denmark Norway, et al. are relatively early, and the published research results are. mainly concentrated around 2016.The research results of China, Turkey, Iraq, Egypt, Iran, Poland, Mexico, Thailand, India, Malaysia, Pakistan, Saudi Arabia, Czech Republic, Brazil, et al. were mainly concentrated after 2018, which was carried out later.

In terms of the number of publications by research institutions, the top ten research institutions are all located in China. These institutions, including *Shanghai Jiao Tong University, Tongji University* and *Fudan University* have established cooperative relationships with each other. *The Massachusetts General Hospital, Harvard University, Huazhong University of Science and Technology* and *Zhengzhou University* also cooperate actively. However, we observed that while research institutions in China publish a large number of research papers, their collaboration with research institutions in other countries is limited. The lack of international cooperation may hinder the long-term development of academic research. While there has been some cooperation with certain countries, it has not been as extensive and close as hoped. Therefore, in order to jointly advance the development of ncRNA in OS, we advise research institutions from various nations to engage in broad collaboration and exchanges.

Since the majority of studies on ncRNA in OS were published in *Oncology Letters* (n = 139, Q3), it is clear that this journal is now the most well-liked in the field. *Biomedicine & Pharmacotherapy* had the largest impact factor (IF = 7.5, Q1), followed by *Cancer Cell International* (IF = 5.8, Q1). According to our analysis, more than half of the co-cited journals are high-impact Q1 journals. These are undoubtedly reputable, international journals that promote ncRNA research in OS. Additionally, very few studies on ncRNA in OS are currently published in journals that are clinically relevant, with the majority of the research being published in journals related to biology, molecular science, and pathology. This shows that the majority of the current research is still in the basic research stage.

The authors with the largest number of publications are *Wei Wang* (n = 33)*, Wei Liu* (n = 32) *and Zhenfeng Duan* (n = 31)*. Wei Wang* published 33 papers, two of which explored the molecular mechanism and pathogenesis of OS through integrated analysis of mRNA and miRNA microarrays and circular RNA 0001785 as a ceRNA to upregulate HOXB2 regulation by sponge miR-1200 (28, 29). Additionally, they discovered that osteoblastoma and OS can be distinguished by hypoxia-related microRNA-210, which is a diagnostic marker (30). Another important discovery is that CircDOCK1 can advance OS and control cisplatin sensitivity via the miR-339-3p/IGF1R axis (31). Therefore, a critical mechanism and potential therapeutic target for OS may be the circDOCK1/miR-339-3p/IGF1R axis. This discovery offers an additional molecular strategy to help the pathological characterization of two difficult-to-diagnose musculoskeletal cancers. 32 papers by *Wei Liu* were published. He demonstrated that miR-221-3p (32) and circular RNA PVT1 (circPVT1) (33) are anticipated to be new targets for OS treatment and that inhibition of miR-210-5p may represent a promising OS treatment (34) in addition to identifying the molecular mechanism of long non-coding RNA (lncRNA) PGM5-AS1 in EMT and OS progression (35). *Zhenfeng Duan* has published 31 related papers. He found that multidrug resistance in osteosarcoma is associated with downregulation of miR-15b, and miR-15b reconstruction can reverse chemotherapy resistance of OS (36). In addition, he also found that miR-199a-3p may play a functional role in OS cell growth and proliferation, and improving the function of miR-199a-3p may provide help for osteosarcoma treatment (37). His findings provide new insights for miRNA research in osteosarcoma. *Wang Y* (cited = 676) is the author with the most citations. In 2009, *Wang Y* proved that the expression of miR-140 was connected to the chemosensitivity of OS xenografts utilizing high-throughput miRNA expression analysis. By blocking HDAC4-mediated G1 and G2 phase arrest, MiR-140 inhibits cell growth and contributes to chemoresistance (38). For the development of novel treatment strategies to overcome drug resistance, MiR-140 may be a viable target. Both the molecular mechanism of miR-215 in chemoresistance in OS cells and the downregulation of microRNA-143 in OS, which promotes apoptosis and reduces tumorigenicity by targeting Bcl-2, were investigated by *Wang Y* in 2010 (39, 40). In OS cell lines, miR-133a was discovered to suppress cell growth, enhance apoptosis, and imply its potential for cancer treatment in 2013 (41). The same year, it was discovered that miR-103 and -107 govern DNA damage repair (42) and that miR-335 prevents OS cell migration by targeting the ROCK1 gene (43), revealing new participants in the progression of cancer and the response to chemotherapy. *Wang Y* made the discovery of the novel long non-coding RNA known as hypoxia-inducible factor 2 promoter upstream transcript in 2015. This RNA inhibits OS stem cells *in vitro*. TUG1 can be employed as a molecular marker to keep monitoring and forecast prognosis because it is connected with OS disease status and a poor prognosis (44).*Wang Y* provided evidence in 2016 that miR-29b suppresses tumor growth in OS by targeting CDK6 during proliferation and migration (45). Clearly, *Wang Y’s* accomplishments have established a theoretical and experimental basis for ncRNA study in OS.

### 4.2 Knowledge base

When a reference is cited by multiple publications together, it is called a co-cited reference. Co-cited reference can be considered as the foundation for studying a particular topic (46). To determine the research foundation of ncRNA in OS, we chose 10 co-cited articles with the highest number of co-cited articles for this bibliometric analysis (**Table 5**; **Figure 8**). *Ottaviani G et al.* published the most co-cited study in 2009, and the research provides methods, knowledge and explanation of the epidemiology of OS, and communicates the results to patients and their families (47). The second co-cited study is about real-time quantitative polymerase RCR and 2(-Delta Delta C(T)) Method of related gene expression published by *Livak KJ et al.* in 2001 (48). Up to now, PCR is still the main tool to study the role of ncRNAs in OS. The third co-cited study is a review published by *Bartel DP* in the journal Cell in 2004, which is the most influential among the 10 articles. According to their research, miRNAs are endogenous 22ntRNAs that target mRNA for cleavage or translation inhibition. MiRNAs are thought to have a significant regulatory role in both plants and mammals. Although miRNAs have just recently come to light, they are a diverse class of molecules that regulate gene expression in multicellular organisms and may have an impact on the production of several protein-coding genes (49). The findings of this study are crucial for understanding how miRNAs are controlled in OS. The fourth co-cited study, published by *Mirabello I et al.* in 2009, gave a detailed description of the incidence and survival of OS and compared age and ethnic groups. Their studies showed significant differences in morbidity, survival, pathological subtypes and anatomical sites among different age groups, and quantified the effects of OS as a secondary cancer on morbidity and mortality in patients with Paget disease (50). The biology and epidemiology of OS may be better understood as a result of these research. The fifth co-cited study was published by *Isakoff MS et al.* in 2015 regarding the current treatment and combination therapy for OS. They believe that high-dose methotrexate, doxorubicin and cisplatin (MAP) are the most effective treatments. The use of extra cytotoxic chemotherapeutic drugs like ifosfamide and the inclusion of biological therapies like cell wall tripeptide did not eventually increase the survival rate of patients with OS (51). It is undeniable that the survival rate of OS has significantly improved after the emergence of multi agent chemotherapy regimens. However, due to the fact that the research data at that time was nonrandom, there is still a lot of controversy about the ideal combination of chemotherapy drugs. The sixth co-cited study was published by *Luetke A et al.* in 2014. They reviewed the progress in the treatment of OS in terms of etiology, genetic instability, extensive histological heterogeneity, lack of biomarkers, local invasiveness and rapid metastatic potential of OS (52).Overall, the following subjects are covered by the top 6 references that were also cited: gene expression, regelation mechanism, survival rate and prognosis, biomarker, metastasis, treatment response, chemotherapy drugs and miRNA signatures, which are the key research of ncRNAs in OS.

### 4.3 Hotspots and frontiers

References with citation bursts are widely cited and mentioned by other scholars, so these literatures suggest new problems in specific research fields (53). The biological function and pathogenicity of miRNA in OS, as well as how to employ miRNA to stop OS migration, are among the core research issues of ncRNA in OS research, as evidenced by the main research contents of the strong citation burst literature (**Table 5**). They include *miR-140, miR-21, mir-34a, etc.* Keywords can aid us in swiftly capturing the distribution and development of ncRNA hotspots in OS research in addition to citing new literature. **Table 6** includes the following keywords: *apoptosis/proliferation, migration and invasion, prognosis, biomarker, chemoresistance*, et al. As shown in **Figure 10**, our analysis of trend topics and keyword clustering shows that the research of ncRNA in OS mainly focuses on the following aspects:

#### 4.3.1 Migration and invasion

There is a ton of evidence suggesting miRNA and lncRNA, two significant members of the ncRNA family, are crucial for the development of OS and cancer (54–56). MiRNA has made significant strides in the regulation of post-transcriptional gene expression in both plants and animals, which is connected to the incidence and growth of OS. On the one hand, miRNA is significant for OS pathogenesis and has diagnostic utility (57). On the other hand, miRNA is also a biomarker for a variety of disorders.

MiR-21 and Mir-34 are important parts of the top ten keywords in miRNA research. MiR-21 has two sides. Exosomes from human umbilical cord blood hasten the healing of skin wounds by enhancing fibroblast and angiogenesis activity via miR-21-3p (58). Simultaneously, miR-21-3p from nicotine-treated macrophage exosomes may quicken the onset of atherosclerosis by enhancing VSMC migration and proliferation via its target PTEN (59). Mir-34a is in charge of the multifunctional regulatory center of the OS network, participates directly or indirectly in the control of several genes, and is critical for controlling OS cell proliferation, differentiation, migration, and apoptosis (60, 61). It might be a crucial OS biomarker with therapeutic and diagnostic implications. Bioengineering miR-34a prodrug and doxorubicin synergistically inhibit the growth of human OS cells through RNA interference and DNA insertion, reduce the protein levels of miR-34a targeting (proto) oncogenes (including SIRT1, c-MET and CDK6), change the process and invasion ability of OS cells, and synergistically inhibit the growth of OS (62). The combination of miR-34a and celecoxib can also achieve similar effects (63).LncRNA and CircRNA are also important parts of the ten research keywords of Non-coding RNAs. lncRNA is involved in the development of a variety of tumors (64, 65), including tumorigenesis, proliferation, migration, invasion, metastasis and angiogenesis (66, 67). It can be used as a potential prognostic factor for cancer by using its therapeutic function in targeted selective treatment mode (68). CircRNA also has two-sidedness. While circ-001422 gene overexpression has the opposite effect, circ-001422 gene deletion dramatically increases the proliferation and metastasis of OS cells while promoting their death (69). At the same time, CircRNA regulates gene expression by binding miRNAs (70, 71). CircDOCK1 works with the miR-339-3p/IGF1R axis to advance OS and control cisplatin sensitivity. Therefore, a critical mechanism and potential therapeutic target for OS may be the circDOCK1/miR-339-3p/IGF1R axis (31).

#### 4.3.2 NcRNAs tumor markers

Biomarkers have become a new prospect due to their advantages of being simple to collect samples, minimum injury to the body, high sensitivity, and specificity (72). OS cannot be effectively identified at an early stage without efficient biomarkers. With the progress of technology, it has become clear that ncRNAs such as cirRNA, lncRNA, and miRNA play a significant role in the incidence and development of cancers. As a result, they can be applied as novel biomarkers for prognosis, diagnosis, and treatment. More and more evidence reveals the role of miRNA as a biomarker of disease. The results showed that circulating levels of tumor suppressors miR-579-3p and miR-4488 predicted progression-free survival (PFS) (73). Several miRNAs, including miRNA-100, miRNA-155, miRNA-21, miRNA-34a, and miR-let-7 are anticipated to be useful noninvasive laryngeal cancer markers (74). Downregulation of miR-145 in tumor tissue or peripheral blood indicates poor prognosis in patients with a variety of malignancies, which is also a biomarker (75). Poor survival in human HNSCC is correlated with increased expression of miR-21, miR-18a, miR-134a, miR-210, miR-181a, miR-19a, and miR-155 (76, 77). Additionally, MiR-21 levels were discovered to be higher in OS patients, with levels being notably high in those who had lung metastases (78). Lnc-meg3, lnc-pvt1 and circ-itch are not only the top ten detailed keywords for studying ncRNAS in OS, but also biomarkers for other diseases. lnc-meg3 may be Biomarkers associated with acute myeloid leukemia (79). As a biomarker, NRC-MEG3 is useful for disease management, treatment optimization, and improved prognosis in pediatric AML (80) and cALL (81) patients. The low expression of circ-ITCH is substantially connected with the aggressive clinicopathological characteristics and poor prognosis of various malignancies, according to research by Xiao-Dong Sun and Da-Wei Sun et al (82). Therefore, circ-ITCH can be used as a molecular therapeutic target and prognostic marker for human cancer. Siyuan Wang and Shengqiang Fu also confirmed that circ-ITCH is associated with better clinicopathological indicators, and circ-ITCH has potential as a biomarker (83).

#### 4.3.3 NcRNAs and drug resistance

One of the biggest obstacles to treating OS is chemotherapy resistance. Mir-143, lnc-meg3 and cir-cpvt1 are also important parts of the ten research keywords of Non-coding mir-143 is regulated by has-circ-0001982 in cells, and regulates malignant behaviors such as proliferation, invasion, migration and multi-drug resistance of OS cells, and the two are negatively correlated (84). Doxorubicin is an antibiotic. It has a wide anti-tumor spectrum and belongs to cycle non-specific drugs. It has killing effect on tumor cells of various growth cycles (85). The transfer of exosome miR-143-3p makes OS cells resistant to adriamycin. miR-143 was used by lncRNA FOXD2-AS1 to inhibit cisplatin resistance in human OS cells in drug-resistant cell lines (86). Lnc-meg3 was adversely correlated with overall survival (OS) and progression-free survival, and it demonstrated excellent sensitivity and specificity in predicting treatment resistance (87). Cir-cpvt1 mediates OS cell proliferation and chemotherapy resistance through the miR-24-3p/KLF8 axis (88). All these provide some reference for clinical prevention of drug resistance in OS cells.

In conclusion, ncRNA not only contributes to the incidence and development of OS as a therapeutic carrier, but also to the development, manifestation, and management of various diseases. On the one hand, the part non-coding RNA plays in the formation and progression of OS can aid in our understanding of its pathophysiology as well as the causes of its occurrence. On the other hand, it also aids in the analysis of the etiology of other associated diseases and helps in the identification of other disease processes. Therefore, the study of ncRNA treatment strategy has important application value for the treatment of OS.

#### 4.3.4 Current hotspots and trends

From the distribution characteristics of keywords in the average publication year, the research on ncRNAs of OS is mainly concentrated in 2017-2020. Among them, the research of *tumor microenvironment, immune, exosome, cerna and bioinformatics* in ncRNA is mainly concentrated in 2020 or later, indicating that they are the research hotspot since 2020. Indeed, the tumor microenvironment, exosomes, and immune responses are closely interrelated. OS thrives within the bone microenvironment, which is an exceedingly specialized, intricate, and highly dynamic milieu composed of bone cells, stromal cells, vascular cells, immune cells, and the extracellular matrix. Under physiological conditions, the coordinated activities of bone, vascular, and stromal cells ensure bone homeostasis through robust paracrine and cellular communication (89). The crosstalk between OS and the microenvironment involves many environmental signals induced by a wide range of cytokines, chemokines and soluble growth factors (90). The tumor microenvironment exists as a network of immune cells. Its functions are diverse and complex and allow tumors to grow within bone by hijacking key physiological pathways that promote survival and proliferation. These functions derive in part from tumor-derived exosomes, which drive the behavior of osteoblasts and create a permissive microenvironment conducive to tumor cell homing (91). In recent years, many studies have confirmed that solid tumor-derived exosomes can contribute extensively to immunosuppression. It has been shown that such exosomes can inhibit the activity of T cells and NK cells, and even induce T cell apoptosis, through multiple pathways to support OS cells in evading immune surveillance, while stimulating the activity of bone marrow-derived suppressor cells (92). A recent study found that exosomal PD-L1 expression was significantly higher in patients with OS lung metastases than in those without metastases (93). They further explored the role of PD-L1-containing exosomes and found an increased rate of lung metastasis after treatment with exosomal PD-L1 in a mouse model. These results suggest that OS cells stimulate lung metastasis by releasing exosomal PD-L1 and that detection of exosomal PD-L1 expression in serum can predict the progression of lung metastasis in OS patients. It has also been found that miR-21 contained in exosomes from cancer cells in the tumor microenvironment may act on cancer cells and the surrounding tumor microenvironment. In human serum and plasma, exosomal miR-21 levels differed between osteosarcoma patients and healthy controls, supporting the role of miR-21 as a biomarker for OS (94). Many studies now suggest that miR-21 target genes are involved in tumor progression. miR-21 may significantly affect cancer cell plasticity, leading to tumor progression, metastasis, angiogenesis, and immune escape in osteosarcoma (95, 96). Lavinia Raimondi et al. (97) investigated miRNAs from exosomes and their parental cells, and identified miRNAs involved in a variety of biological processes and carcinogenesis. Some of these miRNAs are already known for their involvement in the establishment of the tumor microenvironment, such as miR-148a and miR-21-5pa. their study re-emphasizes the importance of OS exosomes in the tumor microenvironment and are packaged by specific miRNAs. Therefore, understanding the biological origin and function of exosomes is valuable for the diagnosis and treatment of cancers, including OS.

Immunotherapy has been shown to be a promising therapeutic strategy against human malignant tumors, and its efficacy has received widespread attention (98). It involves the application of tumor vaccines, immunomodulators, genetically modified T cells, cytokines, immune checkpoint inhibitors, or combination therapies, which can largely reduce therapeutic side-effects, enhance therapeutic efficacy, and improve the quality of life of cancer patients (99). In recent years, a large number of preclinical trials have supported the use of immunotherapy in OS (100). Merchant et al. reported in a pediatric phase I clinical trial that 25% of patients with OS had stable disease and acceptable immune-related adverse events after treatment with ibritumomab (101). However, the main issue regarding ibritumomab treatment in pediatric patients is gastrointestinal toxicity. Therefore, safer and more effective checkpoint inhibitors and immunotherapies are needed in the future. It has also been found that different OS patients have different immune microenvironment characteristics and therefore respond differently to immunotherapy (102). Therefore, studying the immunological characteristics of OS tissues can also help to improve the efficiency of immunotherapy. In summary, challenges remain for immunotherapy, including identifying the most appropriate checkpoints and immunotherapies, reducing the toxicity of cancer vaccines and cytokines, and avoiding paradoxical or excessively progressive disease. Exploring predictive biomarkers is equally important, as this may allow for more personalized immunotherapy for OS.

### 4.4 Advantages and shortcomings

This study offers a number of distinctive benefits. First and foremost, for the first time, we employed bibliometrics to thoroughly examine the non-coding RNA research in OS, which can offer thorough guidance for academics that focus on related research. Secondly, our survey was done using three bibliometric tools, two of which are well-known in the bibliometrics community (VOSviewer and Cite Space), making it more likely that our data analysis was unbiased. Finally, bibliometric analysis offers cutting-edge insights and more thorough hot spots than conventional reviews.

Of course, there are some weaknesses in this study as well. First of all, this study only used data from the Wo SCC database, neglecting data from other databases, which means it might have missed some related research. Second, we reviewed studies that were written in English, which may indicate that non-English writing samples are undervalued.

## 5 Conclusions

NcRNA offers significant potential for use in OS and for research. The quick rise in the number of papers published suggests that academics are placing a greater priority on the study of ncRNA in OS. At present, China has the largest number of publications. But there is a need to improve collaboration and communication across nations, organizations, and writers. More and more works appearing in international core journals demonstrate a major influence. On the one hand, understanding how ncRNA functions in OS development will aid in the analysis of OS causes and the diagnosis of the OS disease process. On the other hand, it also aids in the analysis of the pathophysiology of other connected diseases, aiding in the detection of additional disease processes. From the current point of view, the tumor microenvironment, exosomes and immunotherapy are the research hotspots and trends of ncRNAs in OS.

## Data availability statement

The original contributions presented in the study are included in the article/**Supplementary Material**. Further inquiries can be directed to the corresponding author.

## Author contributions

LC: Data curation, Formal Analysis, Methodology, Visualization, Writing – original draft. LH: Software, Visualization, Writing – original draft. BL: Software, Writing – review & editing. YZ: Data curation, Formal analysis, Writing – original draft. LL: Supervision, Formal analysis, Writing – review & editing. ZW: Funding acquisition, Resources, Writing – review & editing.
